# Online open-book examination of undergraduate medical students – a pilot study of a novel assessment method used during the coronavirus disease 2019 pandemic

**DOI:** 10.1017/S0022215121000141

**Published:** 2021-01-08

**Authors:** S Sarkar, P Mishra, A Nayak

**Affiliations:** 1 Department of ENT, All India Institute of Medical Sciences (‘AIIMS’), Bhubaneswar, Odisha 751019, India; 2 Department of Physiology, All India Institute of Medical Sciences (‘AIIMS’), Bhubaneswar, Odisha 751019, India

**Keywords:** COVID-19 Pandemic, Learning, Medical Education

## Abstract

**Background:**

The coronavirus disease 2019 pandemic has posed a new challenge for medical educators worldwide. While teaching and learning shifted online, assessment posed a roadblock. A pilot study was performed to check the feasibility and acceptability of online open-book examination.

**Methods:**

A pilot study was carried out on sixth semester (fourth year) students. An online open-book examination was conducted on an ENT topic, and feedback was obtained using a pre-validated questionnaire. Two teachers scored and collated the answers, and the marks were averaged for each candidate.

**Results:**

Ninety-eight students appeared for the examination: 21.4 per cent failed and 78.6 per cent passed. Eight students scored above 75 per cent correct. Only 55 students volunteered to give feedback; most agreed that the best advantage of this assessment was that it was stress-free. The disadvantage most complained of was network connectivity issues.

**Conclusion:**

Online open-book examination has the potential to be the new normal in the present circumstances and beyond.

## Introduction

The coronavirus pandemic has created a lot of confusion among medical educators. Medical schools worldwide are searching for effective and innovative methods to conduct classes and examinations online. Many countries, including India, went into a state of lockdown in March 2020, with educational institutions remaining closed to date. Most medical schools have adapted to the situation by shifting to online classes. The challenge for us in our institute was how to assess these students and ensure that they are learning. We also need to validate the scores attained in these online examinations as there is a risk of cheating during a remote online examination when no invigilator is there to monitor examinees. Given the new norms of lockdown and social distancing, the way we assess students needs to be readdressed and rethought.

The online assessment of students comes with its own set of hurdles for teachers, such as how to ensure that students are not cheating, and how to invigilate them online. In order to avoid the drawback of monitoring students during an examination, the medical education unit of this institute decided to try an online open-book examination method. Online assessment and open-book examination have both been utilised previously in medical assessments. An open-book test is documented as being a useful tool for encouraging deep learning and critical thinking in students.^1–3^

No online open-book examination used in medical education had been reported when the article was initially submitted. However, during revision, we found an article on online open-book examination by Eurboonyanun *et al*., published in September 2020.^4^

DiCarlo stated ‘learning is not committing a set of facts to memory but the ability to use resources to find, evaluate, and use information’.^5^ It was essential that the questions in the online open-book examination tested higher-order cognition, in order to check the application of the knowledge the students had acquired. This pilot study aimed to check the feasibility and acceptability of an online open-book examination for medical students.

## Materials and methods

**Fig. 1.**
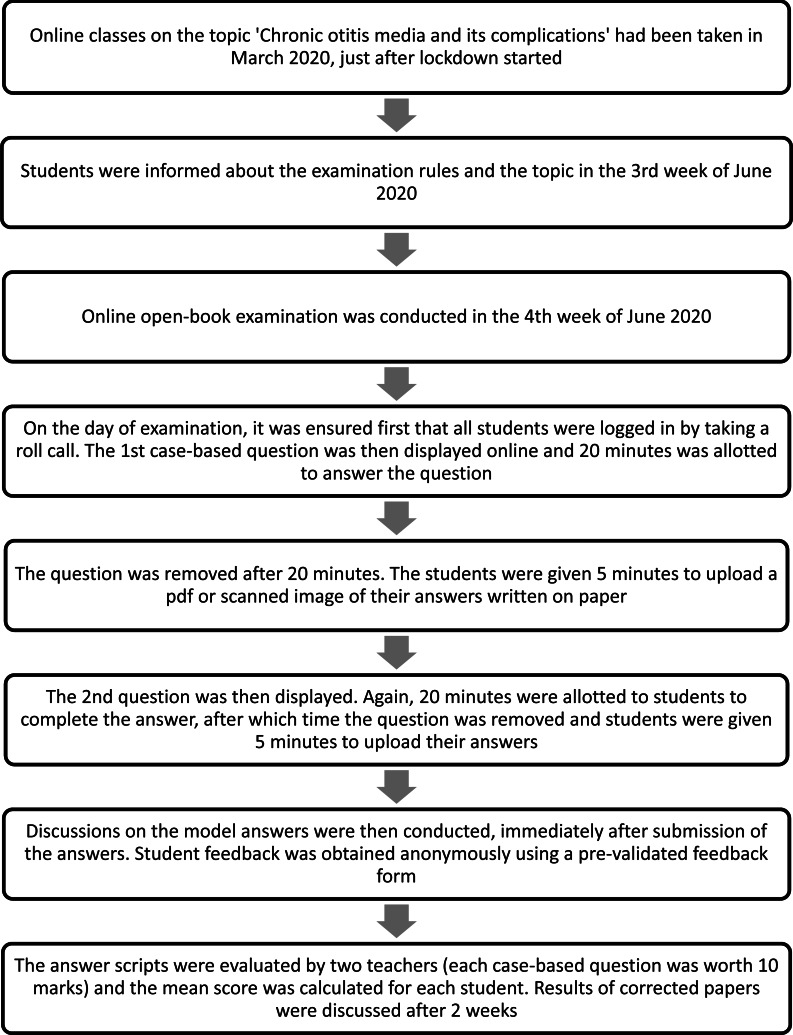
Flowchart showing the conduct of the examination.

The online open-book examination was planned for sixth semester (fourth year) students on the subject of otolaryngology (Table 1). Students were informed about the topic of chronic otitis media and its complications, and the examination rules, one week prior to the test. The conduct of the examination is shown in the flowchart (Figure 1).

**Table 1.** Questions used for open-book examination

Question number	Question text	Total marks
Question 1	A 25-year-old man presented to the ENT out-patient department with the complaint of foul-smelling, scanty blood-tinged discharge from his right ear & occasional episodes of dizziness during the past 6 months. Ten years previously, he had visited an ENT surgeon with profuse discharge from the right ear. He had been diagnosed with a central perforation & was advised surgery which he had refused at that time	10 marks
a.	State the probable diagnosis for his present condition?	1 mark
b.	Explain the theory underlying his transition to the present condition? How will you refute the other theories?	4 marks
c.	What might be the clinical consequences if the patient refuses surgery again?	5 marks
Question 2	A 5-year-old child presented to the emergency department with fever, with chills & rigor, & with profuse sweating following a fall in temperature. This has been occurring once a day for the past 2 days. He has a 2-year history of foul-smelling discharge from his right ear. He lives in a shanty with his ragpicker parents. On examination, he was found to be emaciated, his right ear was filled with foul-smelling pus, his right mastoid & occipital region were oedematous, & the right side of his neck was tender	10 marks
a.	What is the probable diagnosis of the patient?	1 mark
b.	What is the pathophysiological basis of his symptoms & signs? What else can you check clinically to aid your diagnosis?	3 + 1 = 4 marks
c.	How do you think the disease must have spread to cause this condition, & what other complications can it cause? Explain with a diagram	3 + 2 = 5 marks

Feedback was obtained from students (as this was something new that they had not undergone previously) using a validated questionnaire consisting of open-ended and Likert scale based questions on the day of the examination (Table 2).

**Table 2.** Feedback questionnaire

Question number	Question text
1	How easy (1) or difficult (10) did you find the test questions, on a scale of 1 to 10?
2	Are you aware of the open-book examination? Yes / No
3	How did you prepare for the examination?
4	Was the time given to write the answers sufficient? Yes / No
5	How much time did you take to read & write the answers?
6	Did you discuss the answers with friends? Yes / No
7	Should the open-book examination continue? Yes / No
8	Do you want to change anything? Yes / No
9	If Yes, what do you feel needs to change?
10	What are the positive aspects of this (online open-book) examination format?
11	What are the negative aspects of this (online open-book) examination format?
12	As an online examination, which is better? Multiple choice question / Open-book long answer question / Closed-book long answer question or short answer question

Data assimilation and statistical analysis were conducted using MS Excel® spreadsheet software and GraphPad statistical software.

## Results

Ninety-eight students presented for the online open-book examination; two students did not attend. The pass mark for the examination was 50 per cent, which is equivalent to 10 marks (a total of 20 marks could be obtained). Out of 98 students, 21 did not achieve the pass mark (a 21.4 per cent failure rate), while 77 passed the examination (a 78.6 per cent pass rate). This is comparable to the pass percentage of the closed-book examinations, which included a mix of recall and application, and comprehension questions, conducted earlier in the same group of students. Eight students scored above 75 per cent in the examination. The results were validated by two specialists individually. The scores of those students who failed the examination were averaged, to eliminate bias in the correction of papers.

**Fig. 2.**
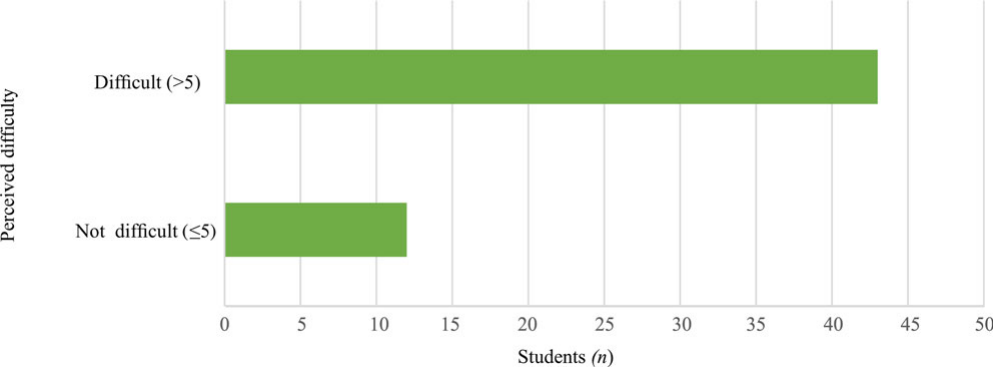
Difficulty of the assessment method, as perceived by the respondents, on a scale of 1 to 10.

Only 55 students volunteered to give feedback for the new examination system, even though it was anonymous. More than half (78.2 per cent) of the respondents considered this method of assessment as difficult (rated on a scale of 1 to 10, whereby a rating of more than 5 represented difficult and a rating of 5 or lower represented not difficult), while 21.8 per cent considered this method of assessment as not difficult (Figure 2).

Twenty-six responders stated that the time allotted for the questions was adequate, and 28 responders said it was inadequate; 1 individual did not respond to the question. Fifteen respondents (27 per cent) said that they consulted with friends while answering the questions, whereas 39 (72.2 per cent) said that they did not consult with their friends during the examination and answered independently; 1 student did not respond to this question. Twelve of the 15 responders who consulted with friends found that the time allotted for answering questions was inadequate, while 16 out of 39 respondents who did not consult friends found the time allocated for answering to be inadequate. A Fisher's exact test revealed a statistically significant effect (*p* < 0.05): the students who consulted with friends during the examination found the time allotted to be inadequate, as they lost time in mutual discussion (Table 3).

**Table 3.** Comparison of students’ responses regarding consultation during examination and perceived adequacy of time allotted

Consultation during examination	Students who responded that they had sufficient time to answer	Students who responded that they did not have sufficient time to answer	Row totals
Students who discussed answers with friends	3	12	15
Students who did not discuss answers with friends	23	16	39
Column totals	26	28	54

Data represent numbers of students. Applying the Fisher exact test, the *p*-value is 0.014, indicating a significant effect.

When the students were asked what they liked about the online open-book examination format, the following responses were given: (1) the clinical case scenarios helped the students analyse information given previously and deduce the diagnosis accordingly (23.6 per cent); (2) they did not feel stressed the night before the examination (students felt calmer and comfortable during the examination, 49.1 per cent); (3) they had the opportunity to evaluate themselves in terms of preparedness on the topic (5.5 per cent); (4) they did not need to memorise, which helped with thorough, in-depth reading, and they could devote more time and energy to understanding concepts (18.2 per cent); (5) the format helped the students to recollect and learn at the same time (9.1 per cent); (6) they had adequate time to prepare thoroughly (10.9 per cent); and (7) the format was only helpful if students were thorough with their book reading (3.6 per cent).

The things that the students did not like about this format of assessment included: (1) insufficient time (27.3 per cent); (2) they copied from the book rather than thinking too much about it (3.6 per cent); (3) it was challenging to scan and upload answers because of device and internet issues (30.9 per cent); (4) multiple choice questions would have been easier to handle (9.1 per cent); and (5) the format was not useful (16.4 per cent).

Forty out of 55 respondents (72.7 per cent) stated that they did not want further online open-book examinations. The main challenges were considered to be internet and logistical issues, rather than the examination itself, which is perhaps why they did not prefer this mode of examination.

Thirty-six of the 55 responders (65.5 per cent) said that multiple choice questions were preferable to the online open-book examination format; only 12 respondents (21.8 per cent) preferred the open-book examination method.

## Discussion

A previous study found that teachers are very concerned about not being able to cover the required content.^5^ In the same study, it was stated that ‘learning is not committing a set of facts to memory but the ability to use resources to find, evaluate, and use information’.^5^ Hence, we need to reduce the amount of information that a student needs to memorise, and focus more on the development of skills needed to apply the acquired knowledge, in order to solve new problems. Then learners can undertake more in-depth learning activities.

The Centre for Teaching and Learning at the University of Newcastle Australia has produced guidelines for open-book examination.^6^ It clearly states that it is a mode of assessment where students can refer to class notes, textbooks or other approved material while answering questions. Students can also be provided with questions before the formal examination or can complete it as a take-home examination. In the prevailing scenario of lockdowns where it is not feasible to conduct examinations for 100 odd students, this method seems to be ideal; it can be carried out with ease and can promote more in-depth learning.

The University of Newcastle Australia guidelines go on to state that traditional examinations promote rote knowledge and a superficial application of knowledge.^6^ The open-book examination, on the other hand, enables teachers to frame questions in a manner that requires students to answer more critically and analytically, thus encouraging higher-order thinking. In traditional examinations, facts and lower-level cognition take precedence over higher cognition levels and more abstract outcomes such as debating, delineation and application.^1^ In fact, it is rather difficult for the educators to frame proper questions for this type of higher cognition examination. The medical educator must consider the higher-order cognition of the Bloom's taxonomy^7^ while preparing the items, and use appropriate verbs for the questions.

In the current age when practitioners’ knowledge must be up to date, with information at their fingertips and a device in every pocket, it is unprofessional to rely on fallible human memory for the recall of critical facts in the course of patient care.^8^ As teachers, it is stressed that facts are relatively less important, and we are interested in developing students’ higher-order thinking. Open-book examination as a method of assessment reflects the real-life scenario where students are expected to answer problems and questions using ambient knowledge.^8^

A systematic review of non-medical literature found that there are significant benefits to the open-book examination method.^9^ In that study, the open-book examination was perceived to be less stressful for students to revise for, more authentic to clinical practice where information is freely available during a consultation, encourages more in-depth learning and enables assessment of higher-level outcomes.^9^ This is congruent with the feedback given by the students in our study also. The systematic review found that the downsides of the open-book examination were: it takes longer to answer the questions, the answers are more challenging to write, and collaboration between candidates and with others goes undetected. Our study showed that students who were discussing and collaborating during the examination found the time allotted for answering questions to be insufficient; this was a statistically significant finding.

Another study suggested that open-book examination may encourage students to take greater ownership of the learning process, which is within the principles of self-directed adult learning.^2^ Burnout and resilience are the fallouts of any assessment. By altering the assessment method, the stress induced may be more useful in developing the resilience needed for medical practice.^10^ In our study, the students’ feedback suggested they were less stressed in the open-book examination. They indicated that they spent more time understanding the topic rather than just memorising it – recollecting and learning at the same time – and admitted that they would not be able to write answers for chapters that had not been read through thoroughly.

Despite agreeing that the online open-book examination was a good way of assessing their knowledge with less stress, the students aim to qualify for a specialty course for which one should be familiar with multiple choice questions. Hence, the obvious choice for the students is to be tested with multiple choice questions in order to prepare for the entrance specialty examination. Of students, 72.7 per cent replied that they would not prefer the open-book examination method in future, despite appreciating the stress reduction and analytical questions. This reluctance may also have been because of internet connectivity and logistical issues faced during the examination. Eurboonyanun *et al*. (2020)^4^ reported similar findings, in which students preferred closed-book examinations over an online open-book examination. However, they agreed that this format of examination was most appropriate for the current pandemic situation, to prevent the spread of contagion.

- Assessment of medical students has become a major challenge in the existing pandemic scenario
- Open-book examination is a well-known means of assessment practised in many universities
- It is less stressful for students and can assess students’ higher cognition levels
- Formulating appropriate questions is the most important aspect of this type of examination
- The results of online open-book examination replicate students’ scores in a normal examination
- Online open-book examination has potential to be the new normal means of assessing medical students, but is dependent on an adequate information technology infrastructure

The real challenge is convincing the medical educators, who may not like the change and would instead prefer to follow the existing tried and tested system. However, given the challenges presented by the pandemic, online teaching, learning and assessment may be the new normal. A change in assessment philosophy may result in engaged students with more profound and thorough knowledge.

The open-book testing format mirrors the discipline of family medicine, where practitioners refer to written material for clinical decision-making.^2^ Studies of open-book testing have revealed some important outcomes, including reduced examination stress, decreased rote memorising of facts, lasting memory and more constructive student preparation.^11^ Some of these points are mentioned in the students’ feedback for this study.

## Conclusion

The challenges for medical educators presented by the coronavirus pandemic have forced us to search for strategies to continue with our teaching and learning. While online teaching and learning has taken over the regular classes, medical educators are still searching for the perfect online assessment method; this method should be easy to implement and should replicate pre-coronavirus assessment results. Based on the findings of this study, we may conclude that online open-book examinations have the potential to be the new normal in the present circumstances and beyond.
